# The Uncovered Function of the *Drosophila GBA1a*-Encoded Protein

**DOI:** 10.3390/cells10030630

**Published:** 2021-03-12

**Authors:** Or Cabasso, Sumit Paul, Gali Maor, Metsada Pasmanik-Chor, Wouter Kallemeijn, Johannes Aerts, Mia Horowitz

**Affiliations:** 1 Shmunis School of Biomedicine and Cancer Research, Faculty of Life Sciences, Tel Aviv University, 69978 Ramat Aviv, Israel; orcabasso@mail.tau.ac.il (O.C.); zoomitpal@gmail.com (S.P.); galifit@gmail.com (G.M.); 2 Bioinformatics Unit, Faculty of life Science, Tel Aviv University, 69978 Ramat Aviv, Israel; metsada@tauex.tau.ac.il; 3 Medical Biochemistry, Leiden Institute of Chemistry, Faculty of Science, Leiden University, 2333 CC Leiden, The Netherlands; w.kallemeijn@imperial.ac.uk (W.K.); j.m.f.g.aerts@lic.leidenuniv.nl (J.A.)

**Keywords:** *GBA1*, acid β-glucocerebrosidase, Gaucher disease, unfolded protein response, inflammation

## Abstract

Human *GBA1* encodes lysosomal acid β-glucocerebrosidase (GCase), which hydrolyzes cleavage of the beta-glucosidic linkage of glucosylceramide (GlcCer). Mutations in this gene lead to reduced GCase activity, accumulation of glucosylceramide and glucosylsphingosine, and development of Gaucher disease (GD). *Drosophila melanogaster* has two *GBA1* orthologs. Thus far, *GBA1b* was documented as a *bone fide* GCase-encoding gene, while the role of *GBA1a* encoded protein remained unclear. In the present study, we characterized a mutant variant of the fly *GBA1a*, which underwent ERAD and mildly activated the UPR machinery. RNA-seq analyses of homozygous mutant flies revealed upregulation of inflammation-associated as well as of cell-cycle related genes and reduction in programmed cell death (PCD)-associated genes, which was confirmed by qRT-PCR. We also observed compromised cell death in the midgut of homozygous larvae and a reduction in pupation. Our results strongly indicated that *GBA1a*-encoded protein plays a role in midgut maturation during larvae development.

## 1. Introduction

Human acid β-glucocerebrosidase (GCase) is encoded by the *GBA1* gene, mutations in which lead to reduced GCase activity, accumulation of glucosylceramide (GlcCer) and glucosylsphingosine (GlcSph), and development of Gaucher disease (GD). GD is the most common lysosomal storage disorder (LSD), with more than 700 *GBA1* associated mutations (300 mutations were published [1]), and 739 mutations appear in the gnomAD browser (gnomad.broadinstitute.org).

In *Drosophila*, there are two *GBA1* orthologs, *GBA1a* (CG31148) and *GBA1b* (CG31414); both are located on chromosome 3. They are ~2 Kb (3R: 23,700,621–23,702,605) and ~4 Kb in size (3R: 23,704,804–23,708,512), respectively, and are separated by a non-relevant gene (CG31413) (FlyBase.org). The sequences of *GBA1a* and *GBA1b* encoded proteins share ~50% similarity with the human GCase, and the two catalytic amino acids, which determine GCase activity, are identical between human GCase and fly *GBA1a* and *GBA1b* proteins (E235 and E340 in human GCase [2], E298 and E405 in both fly *GBA1*-encoded proteins). The same is true for five of the six amino acids that stabilize the substrate in the active pocket of GCase (Table 1). While expression of *GBA1a* is mostly restricted to bodies, *GBA1b* mRNA is the major species expressed in heads (FlyBase.org) [3,4,5].

Several publications have already described the consequences of mutations in the *GBA1b* gene, which ultimately indicated that it encodes a *bona-fide* lysosomal GCase [3,4,5,6,7].

Despite the high similarity between the sequences of the two *Drosophila GBA1* proteins, it is still unclear whether *GBA1a* protein has any GCase-like activity. Kinghorn et al. [3] found that deletion of *GBA1a* did not affect GCase activity nor substrate accumulation. Additionally, in *GBA1a* RNAi knock-down (KD), no change was observed in climbing abilities or in lifespan of the flies [6]. In a previous study, we documented that flies, expressing a *GBA1a* protein lacking 33 C-terminal amino acids, showed no reduction in GCase activity and no substrate accumulation in either bodies or heads [5]. On the other hand, other publications documented GCase activity, encoded by the *GBA1a* ortholog. Thus, Suzuki et al. [7] observed 91% reduction in GCase activity in brains of flies expressing RNAi against *GBA1a*. Davis et al. [4] noted almost normal *GBA1a*-encoded-GCase activity in bodies of flies, which had no *GBA1b* expression and a 33 C-terminal amino acids deletion of their *GBA1a* encoded GCase.

Interestingly, KD of *GBA1a* led to a delay in midgut development due to delayed autophagy-mediated cell death (autosis), indicating a role for *GBA1a* in midgut development [8].

**Figure 1:**
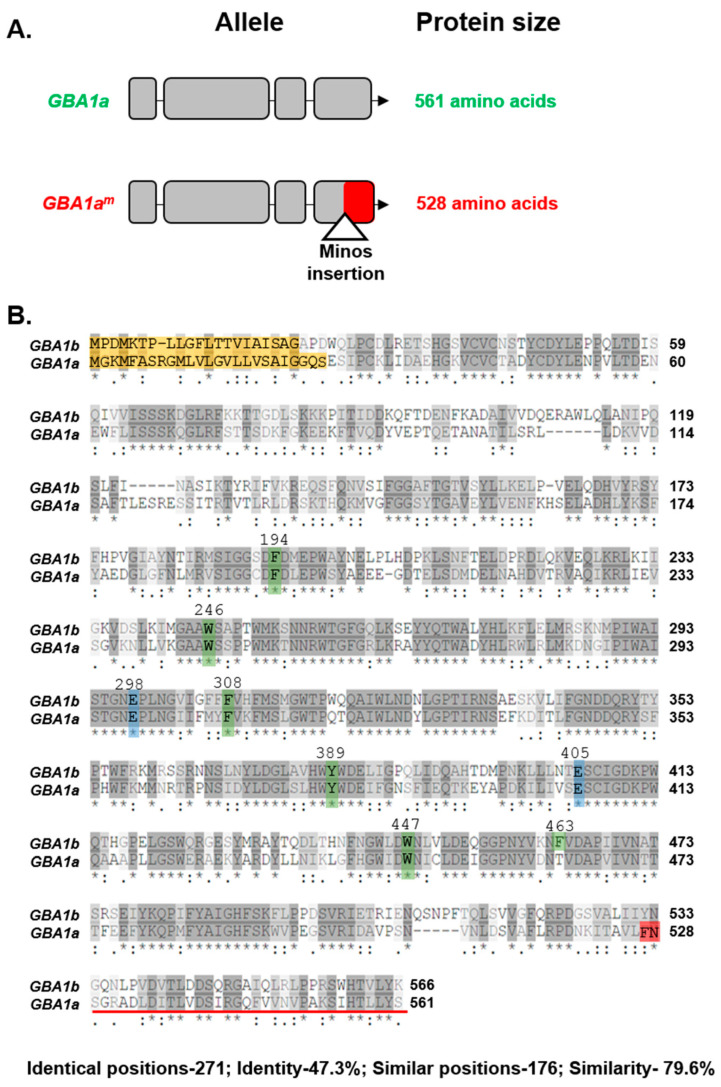
Schematic representation of the *GBA1a* variants and their sequence. (**A**) Schematic representation of normal (upper panel) and mutant (lower panel) *GBA1a* genes. The mutant allele is the outcome of a Minos transposable element insertion in the fourth exon, resulting in a 33 C-terminal amino acids deletion. The missing part of the gene is labeled with red. (**B**) Alignment between *GBA1a* and *GBA1b* encoded protein sequences. Yellow box—predicted signal sequence; green box—amino acids associated with substrate recognition; blue box—amino acids comprising the active site; red box—position of Minos insertion. Red line represents 33 C-terminal amino acids that are missing in the mutant protein. “*”-residue identity; “.”-residue similarity.

To further unravel the role of *GBA1a* in *Drosophila*, and to test whether it has a function in development progression, we used a *GBA1a* mutant line that carries a Minos transposable element in this gene, such that the mutant protein lacks 33 C-terminal amino acids (Figure 1A,B).

Our results indicated that mutant *GBA1a*-encoded protein mildly activated UPR, upregulated inflammation, and downregulated PCD-related genes, which culminated in retarded midgut maturation during early pupation.

## 2. Materials and Methods

### 2.1. Antibodies

The following primary antibodies were used in this study: mouse monoclonal anti-myc antibody (1:1000 for WB; Cell Signaling Technology, Inc., Denver, MA, USA); rabbit polyclonal anti-Erk antibodies (Santa Cruz Biotechnology, Santa Cruz, CA, USA). The secondary antibodies used were: Horseradish peroxidase-conjugated goat anti-mouse antibodies (1:5000 for WB; Jackson ImmunoResearch Laboratories, West Grove, PA, USA) and horseradish peroxidase-conjugated goat anti-rabbit antibodies (1:10,000 for WB; Jackson ImmunoResearch Laboratories, West Grove, PA, USA).

### 2.2. Construction of Plasmids

To create plasmids expressing myc-tagged, normal or mutant *Drosophila* *GBA1a* variants in mycHispcDNA4 plasmid (Invitrogen Life-Technologies, Carlsbad, CA, USA), pUASTmycHis-*GBA1a* and pUASTmycHis-*GBA1a*^m^ [9] plasmids were digested with EcoRI and XhoI and the myc-His containing inserts were cloned between the EcoRI and the XhoI sites of pcDNA4, as previously described [5].

### 2.3. Cells and Transfections

HEK293T cells (ATCC^®^ CRL-11268™) were grown in Dulbecco’s Modified Eagle’s Medium (DMEM; Gibco BRL, Waltham, CA, USA), supplemented with 10% FCS (Biological Industries, Beit-Haemek, Israel) at 37 °C, in the presence of 5% CO_2_. Cells were transfected using calcium phosphate solutions, as described elsewhere [10].

### 2.4. Fly Strains

Fly Strains were maintained on standard cornmeal-molasses medium and kept at 25 °C. All experiments were performed in isogenic *w^1118^* background (which was also used as a control) (Bloomington *Drosophila* Stock Center, Indiana University, Bloomington, IN, USA). Strains harboring a Minos transposable element in *GBA1a* (Mi{ET1}CG31148) or *GBA1b* (Mi{ET1}CG31414) were obtained from Bloomington Stock Center (Bloomington, IN, USA) (Nos. 23602 and 23435, respectively). The balanced lines used in this study were: *w^1118^*;Sco/Cyo;*GBA1a^m^*/TM6b,Sb (*GBA1a^m/+^*) or *w^1118^*;Sco/Cyo;*GBA1a^m^*/*GBA1a^m^* (*GBA1a^m/m^*).

### 2.5. MG132 (Carbobenzoxy-L-Leucyl-L-Leucyl-L-Leucinal) Treatment

HEK293T cells were treated with 15 mM of MG132 (Calbiochem, San Diego, CA, USA) for 20 h.

### 2.6. RNA Preparation

For RNA extraction from flies, adult flies were frozen in liquid nitrogen and then homogenized in TRIzol^®^ Reagent (Life Technologies, Carlsbad, CA, USA), according to the manufacturer’s instructions.

For RNA extraction from larval midgut, animals were collected at the beginning of puparium formation. Four hours later (+4 h RPF), guts were dissected in cold PBS and transferred into 200 µL TRIzol^®^ Reagent containing tubes.

### 2.7. RT-PCR

One microgram of RNA was reverse-transcribed with MMLV reverse transcriptase (Promega Corporation, Madison, CA, USA), using oligo-dT primer (Integrated DNA Technologies, Coralville, Iowa, USA) in a total volume of 25 µL, at 42 °C for 60 min. Reactions were stopped by incubation at 70 °C for 15 min.

### 2.8. Quantitative Real Time PCR (qRT-PCR)

Two microliters of cDNA were used for real time PCR. PCR was performed using the “power SYBR green QPCR mix reagent” kit (Applied Biosystems, Foster City, CA, USA), by Rotor-Gene 6000. The reaction mixture contained 5 µL of SYBR green mix, 300 nM of forward primer, and 300 nM of reverse primer, in a final volume of 10 µL. Thermal cycling conditions were: 95 °C (10 min), and 40 cycles of: 95 °C (10 s), 60 °C (20 s), and 72 °C (20 s). Relative gene expression was determined by Ct value and normalized to that of *rp49* gene. All primers used for the analyses are detailed in Table 2.

### 2.9. Transcriptomic Sequencing and Analysis

The Illumina RNA sequencing was performed at the Crown Institute for Genomics, the Weizmann Institute of Science, Rehovot, Israel. Briefly, cDNAs were prepared from RNA samples, extracted from bodies and heads of 12-day-old flies using in house protocol. For each line (*w^1118^*, *GBA1a^m/+^*, *GBA1a^m/m^*), triplicates of fifty flies each were used. Samples were sequenced on two lanes of Illumina HiSeq 2500 machine, using the Single-Read 60 protocol. The output was ~15 million reads per sample. Reads were trimmed using cutadapt (https://cutadapt.readthedocs.io/en/stable/) and mapped to *Drosophila* melanogaster BDGP6 genome (downloaded from Ensembl genomes) using STAR v2.4.2a [11] [(https://code.google.com/archive/p/rna-star/) default parameters]. Counting proceeded over genes annotated in Ensembl release 31 (http://metazoa.ensembl.org/Drosophila_melanogaster/Info/Index) using htseq-count (https://htseq.readthedocs.io/en/release_0.11.1/) (intersection-strict mode). Differential expression analysis was performed using DESeq2 [12] (doi:10.1186/s13059-014-0550-8) with the betaPrior, cooksCutoff and independentFiltering parameters set to False. Raw P values were adjusted for multiple testing using the procedure of Benjamini and Hochberg [13]. Pipeline was constructed using Snakemake (https://snakemake.readthedocs.io/en/v3.9.1/). Gene lists were created by filtering the genes based on an absolute linear fold change ≥ 2, *p* ≤ 0.05, and reads ≥ 30. To view gene lists as a heat map, the Morpheos tool was used (https://software.broadinstitute.org/morpheus/). Gene lists were analyzed for enriched pathways using the GO enrichment analysis tool (http://geneontology.org) (The Gene Ontology Consortium, CC-BY 4.0). All programs were accessed between: March 2017–September 2020.

### 2.10. Labeling with Activity-Based Probes (ABPs)

Fifty micrograms of protein, extracted from bodies or heads of flies, were incubated with ME569 epoxide (0.5 µM for identification of GCase activity), β-aziridine JJB367 (0.5 µM for identification of GBA1, GBA2, GBA3 activity), TB474 (1 µM for identification of α-galactosidase activity), TB652 (1 µM for identification of β-galactosidase activity), TB482 (3 µM for identification of α-mannosidase activity), TB434 (1 µM for identification of β-mannosidase activity), JJB383 (0.5 µM for identification of α-glucosidase activity) or JJB392 (0.5 µM for identification of β-glucuronidase activity), synthesized at the Department of Bio-Organic Synthesis at Leiden University, as described elsewhere [14,15,16,17,18,19,20], in McIlvaine’s buffer at pH 5.0 (150 mM citric acid−Na2HPO4, pH 5.0) for 30 min at 37 °C. The samples were electrophoresed through 10% SDS-PAGE. Gels were developed by Typhoon FLA 9500 scanner (GE Healthcare, Little Chalfont, UK), capturing Alexa647/Cy5, using PMT 750 V and 100 μm pixel size or by Amersham imager 600 (Amersham, Buckinghamshire, UK).

### 2.11. Measurement of Body Fluid Volume

To measure the body fluid volume, 100 adult females were anesthetized, decapitated, and perforated at their thorax, using insect pins (0.15 mm, Fine Sciences Tools, Heidelberg, Germany). The flies were collected in 0.2 mL PCR tubes. Each PCR tube contained no more than 50 flies. A small aperture was made at the bottom of the PCR tube with a needle, after which it was placed in a 1.5 mL Eppendorf tube and centrifuged at 10,000× *g* for 10 min at 4 °C. The fluid was collected in 1.5 mL Eppendorf tubes and the volume was measured with a Hamilton syringe (Hamilton Company, Reno, NV, USA).

### 2.12. Midgut Morphology and Quantification

Animals were staged and collected at the beginning of puparium formation (0 h relative puparium formation; RPF) and four hours later (+4 h RPF). Midguts were dissected in PBS, fixed in 4% paraformaldehyde in PBS, mounted onto glass slides using Fluorescent mounting medium (GBI labs, Bothell, WA, USA) and imaged using a fluorescent Olympus IX53 microscope (Olympus, Tokyo, Japan) with no filters. Digital images were obtained using cellSens Entry software (Olympus, Tokyo, Japan). Measurements of gastric caeca size were performed using ImageJ software for 10–12 midguts per genotype. Gastric caeca edges were traced and pixel densities were determined using the histogram tool.

### 2.13. Developmental Regulation

For assessing survival rate during development, ten, third instar larvae from each line, were collected into a new cornmeal-molasses medium containing tube, held at 25 °C or at 29 °C. For calculating the percentage of pupae formation, the number of pupae from each line was counted at day four after the initial collection and their number was divided with that of larvae in each tube. For calculating the percentage of enclosed adult flies, their number from each line was counted at day eight after the initial collection and was divided by that of the pupae in each tube. For each line, 10–15 tubes were analyzed at 25 °C and at 29 °C.

### 2.14. Climbing Assay

Climbing behavior of adult flies was measured using a countercurrent apparatus, essentially as described elsewhere [21]. Briefly, groups of approximately 30 flies (both males and females) were given 10 min to adapt in the starting tube, which can slide along the apparatus and then 20 s to move upwards against gravity to the upper frame’s tube. The top frame of tubes was then shifted to the right so that the start tube comes into register with a second bottom tube and flies, which successfully climbed up, were tapped down again, falling into tube 2. The upper frame was then returned to the left and the flies were once again allowed to climb into the upper tube. After five runs, the number of flies in each tube was counted. For each time point, at least four cohorts from each genotype were scored. The Climbing Index (CI) was calculated using the following formula: CI (the weighted mean) =  Σ(mnm)/N. CI ranges from 1 (min) to 6 (max).

### 2.15. Survival Assay

For each fly strain, ten vials, each containing five males and five females, were maintained on food from day one post-eclosion. Fresh food was supplied every other day and deaths were recorded. Kaplan-Meier analyses were performed.

### 2.16. Egg-Laying Experiment

Virgins and males were collected and kept on standard molasses-cornmeal media at 25 °C. Each vial contained females and males from the same genotype (i.e., males and females of *GBA1a^m/m^* and *w^1118^*). Four to five days later, ten males and ten females of each genotype were transferred to five mm, 4% sucrose agar plates with additional sprinkled yeast paste. Plates were cupped by perforated plastic chamber for two hours, followed by eggs counting and transferred to new five mm agar plates. 24 h later, the number of hatching larvae was counted.

### 2.17. Statistical Analysis

Data are expressed as mean ± standard error of the mean (SEM). Unpaired *t*-test (two-tailed *p* value) was used for comparisons of means between the two groups (control versus *GBA1a^m/m^* or control versus *GBA1a^m/+^*). Microsoft Office Excel 2016 was used for all statistical calculations. Values with *p* < 0.05 were considered significant. Kaplan-Meier analysis was performed using XLSTAT software (Addinsoft, NY, USA, 2020.4).

## 3. Results

### 3.1. Activity of GBA1a-Encoded Protein

**Figure 2:**
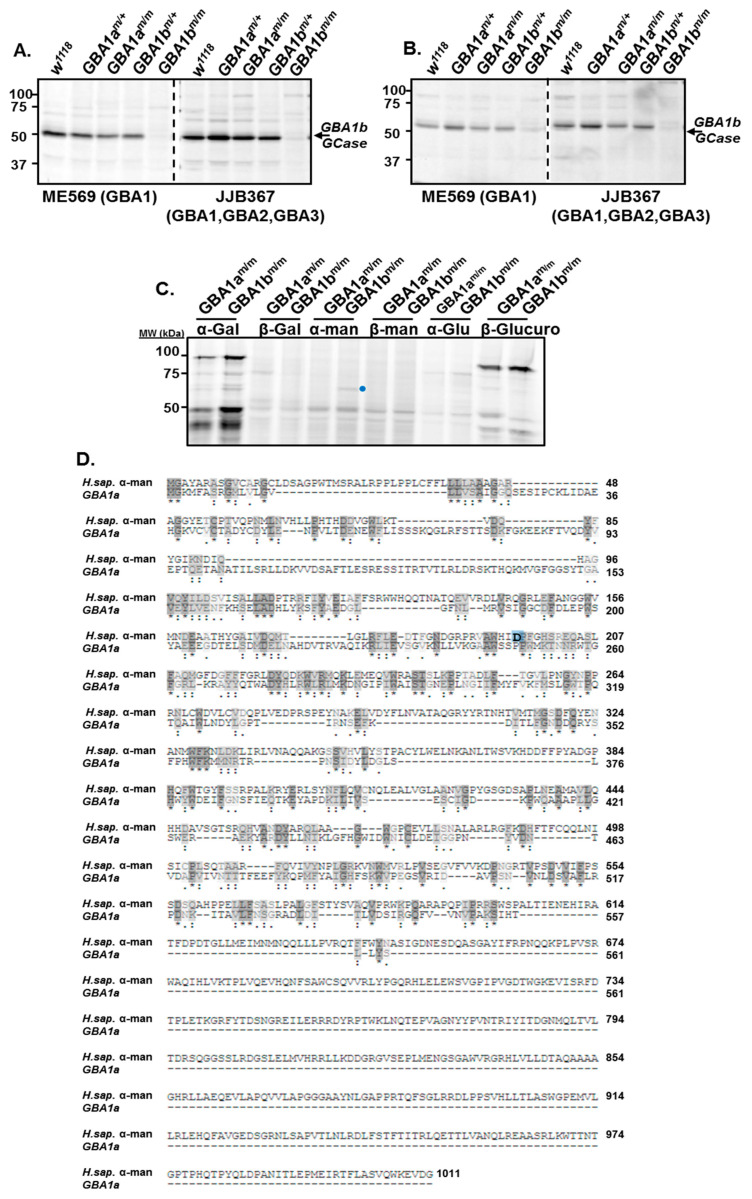
Activity of *GBA1a*-encoded protein. (**A** + **B**) Lysates, prepared from bodies (**A**) or heads (**B**) of homozygotes and heterozygotes: *GBA1a^m/m^*, *GBA1a^m/+^*, *GBA1b^m/m^* and *GBA1b^m/+^* flies and of their age matched controls (*w^1118^*), were incubated with a general GBA (GBA1, GBA2, and GBA3) ABP (JJB367) or a specific *GBA1* ABP (ME362), after which they were resolved through SDS-PAGE. The gel was visualized as indicated in Materials and Methods. (**C**) Lysates prepared from bodies of *GBA1a^m/m^* and *GBA1b^m/m^* flies, were incubated with different activity-based probes: α-galactosidase (TB474), β-galactosidase (TB652), α-mannosidase (TB482), β-mannosidase (TB434), α-glucosidase (JJB383), and β-glucuronidase (JJB392) after which they were resolved through SDS-PAGE. The gel was visualized as indicated in Materials and Methods. Blue dot represents a putative *GBA1a*-specific band. (**D**) BLAST analysis comparing between the human α-mannosidase (H.sap. α-man) and *GBA1a* proteins. Amino acid D196 is the nucleophile of the human α-mannosidase catalytic site and highlighted in blue. “*”-residue identity; “.”-residue similarity.

Multiple sequence alignment of *Drosophila* *GBA1a* and *GBA1b* proteins revealed 47% identity and 80% similarity between them, with a 100% identity between the amino acids constituting their active site (Table 1, Figure 1B). Nevertheless, we could not detect a *GBA1a*-encoded GCase activity using a GCase specific substrate or GCase-specific fluorescently labeled ABP [5] that covalently and irreversibly interacts with *GBA1*-encoded β-glucosidase and can be followed on SDS–PAGE [15]. *GBA1a*-encoded protein did not present any beta glucosidase activity (GBA1, GBA2, GBA3), analyzed using β-aziridine JJB367 [14] as a general probe to detect GBA-encoded protein (Figure 2A,B). To test the possibility that *GBA1a*-encoded protein has another activity, we used fluorescently labeled ABPs specific for different active glycosidases. Lysates prepared from *GBA1a^m/m^* and *GBA1b^m/m^* flies (*GBA1b^m/m^* flies have no *GBA1b*-encoded GCase activity, therefore only *GBA1a*-encoded activity can be tested in them), were incubated with ABPs specific for: α-galactosidase (TB474) [16], β-galactosidase (TB652) [20], α-mannosidase (TB482) [17], β-mannosidase (TB434) [20], α-glucosidase (JJB383) [18], and β-glucuronidase (JJB392) [19] (Figure 2C). The results indicated a possible interaction of a 61 kDa protein in the *GBA1b*^m/m^ derived lysate but not in the *GBA1a^m/m^* derived lysate with the α-mannosidase specific ABP. The molecular weight coincided with the predicted molecular weight of *GBA1a*-encoded mature protein (http://www.bioinformatics.org/sms/prot_mw.html). BLAST analysis (https://www.uniprot.org/blast/) (EMBL-EBI, Cambridge, UK, May 2018) disclosed 25% similarity between *GBA1a* and α-mannosidase encoded proteins (Figure 2D). Therefore, we concluded that the band observed in the *GBA1b^m/m^* derived lysate is not α-mannosidase. Taken together, the results argued that the *GBA1a* derived protein does not have any of the tested glycosidase activity.

### 3.2. ERAD and UPR Activation in GBA1a^m/m^ Flies

**Figure 3:**
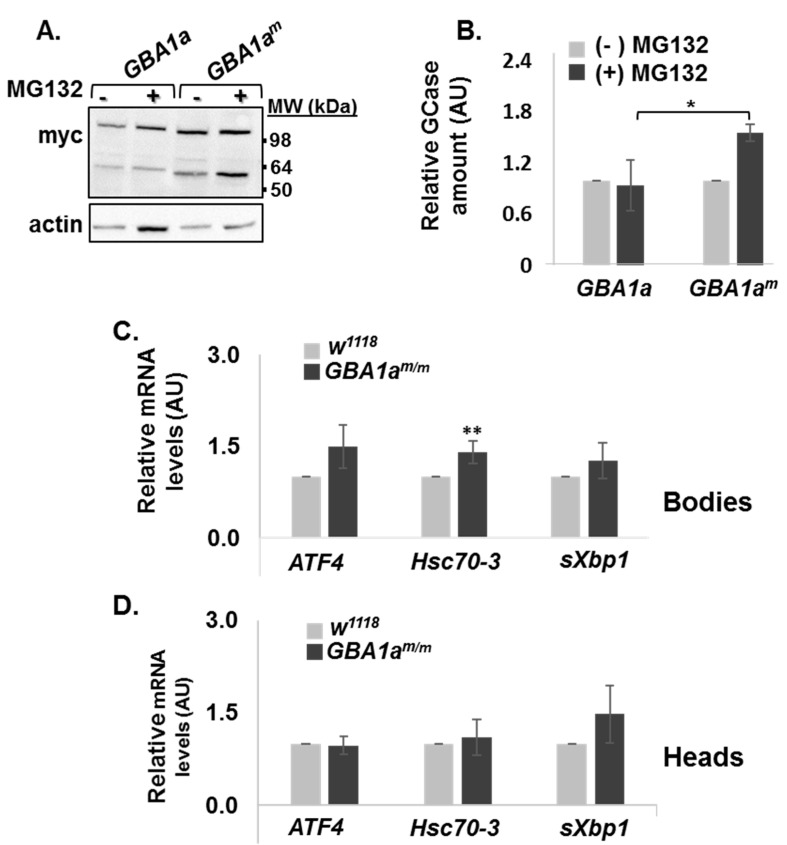
ERAD and UPR of *GBA1a^m^*-encoded protein. (**A**) Protein lysates prepared from HEK293T cells, transfected with plasmids expressing wt or mutant *GBA1a* encoded proteins coupled to a myc-tag were treated or untreated for 18 h with MG132, after which they were resolved through SDS-PAGE. The corresponding blot was interacted with anti-myc or anti actin antibodies, as a loading control. (**B**) To quantify the results, wt and mutant myc-*GBA1a* band intensity was divided by that of actin in the same lane, and the value obtained for *GBA1a* without treatment was considered one. The results represent the mean ± SEM of three independent experiments. (**C** + **D)** mRNA levels of UPR markers: activating transcription factor 4 (*ATF4*) Heat shock-70-3 (*Hsc70-3*) and spliced x-box binding protein (*Xbp1*), in bodies (**C**) and heads (**D**) of *GBA1a^m/m^* and *w^1118^* (control) and flies at 18 days post-eclosion, as analyzed by qRT-PCR. In case of spliced *Xbp1*, the forward primer could anneal only to the spliced form of *Xbp1* mRNA. AU—Arbitrary units. * *p* < 0.05. ** *p* < 0.01.

The results did not reveal any lysosomal function for *GBA1a*-encoded protein; however, we tested whether its mutant form leads to activation of UPR and of ERAD, like the fly *GBA1b*^m^ ortholog [5] and the human *GBA1*-encoded GCase [22,23,24]. With no available anti *GBA1a*-encoded protein antibodies and with difficulties to grow flies in the presence of a proteasome inhibitor, we tested ERAD of *GBA1a*-encoded protein in transfected HEK293T cells expressing the normal or the mutant variants of *GBA1a*, treated with the proteasome inhibitor MG132 (Figure 3A,B). The results indicated 70% increase in the amount of mutant *GBA1a* encoded protein (GBA1a^m^) in the presence of the proteasome inhibitor, arguing that GBA1a^m^ protein undergoes ERAD. As expected, *GBA1a*-encoded protein appeared as two peptides, the upper one represents most probably a dimer, that is unstable under strong reducing conditions [5].

To test UPR activation in *GBA1a^m/m^* flies, mRNA levels of the fly BiP ortholog (Hsc70-3) [25] and ATF4 [26] as well as splicing of Xbp1 [27] were tested by qRT-PCR (CHOP does not exist in *Drosophila*). The results indicated a mild and significant elevation in the expression level of Hsc70-3 in bodies of 18-day old *GBA1a^m/m^* flies (Figure 3C). We did not observe a significant increase in UPR-related genes in heads of *GBA1a^m/m^* flies (Figure 3D), as expected, since *GBA1a* is hardly expressed in heads (http://flybase.org/) [3,5].

Taken together, the results reflected mild UPR activation, a pathological condition triggered by the expression of mutant *GBA1a*^m^-encoded protein in the ER.

### 3.3. Inflammation in GBA1a^m/m^ Flies

Activation of inflammation is well documented in LSDs [28,29,30,31]. This could result from the lysosomal storage of undegraded substrate. However, other processes, like UPR, may lead to inflammation [26,32,33,34]. Since we documented mild UPR activation in the *GBA1a^m/m^* flies, we tested possible inflammation in these flies.

**Figure 4:**
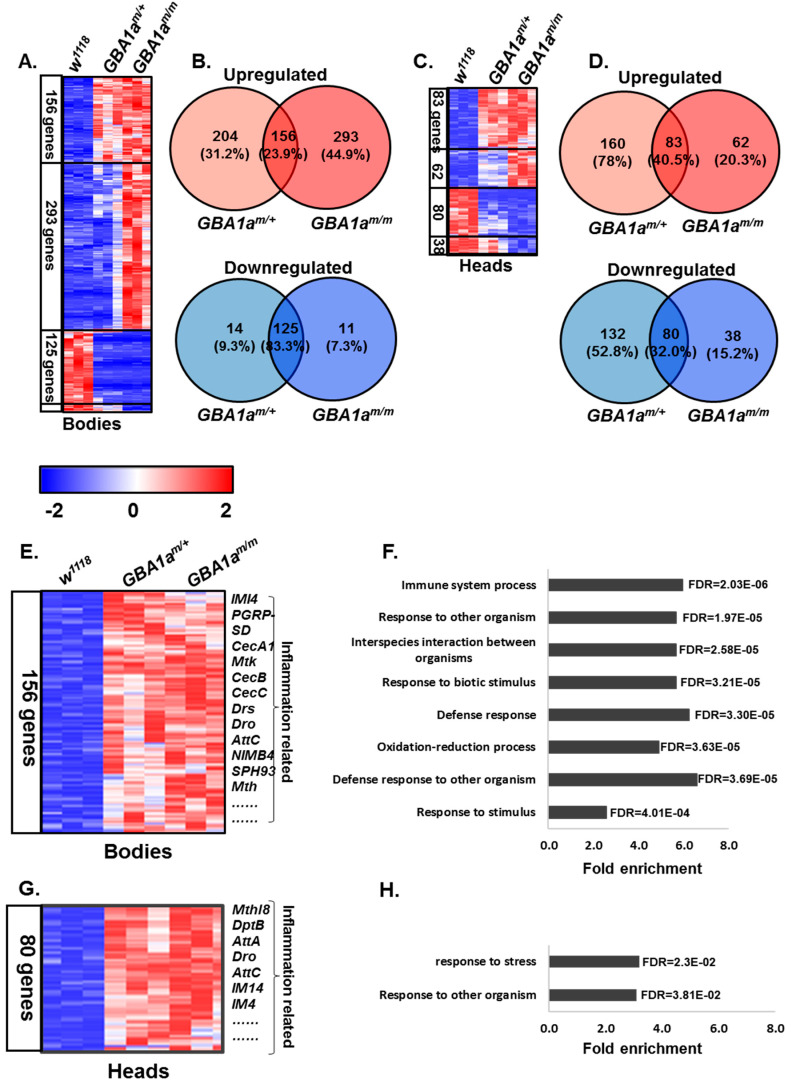
Immune response-related pathways enrichment in *GBA1a^m/m^* flies. (**A** + **C**) Heat maps of differentially expressed genes in bodies (**A**) and heads (**C**) of 12 days old *GBA1a^m/m^* flies, as collected from transcriptomic analysis. The number of genes in each cluster (separated with a black line) is denoted in the left. (**B** + **D**). Venny tool analysis (https://bioinfogp.cnb.csic.es/tools/venny/), showing the number of upregulated (upper panel, red) and downregulated (lower panel, blue) genes in bodies (**B**) and heads (**D**) of *GBA1a^m/m^* and *GBA1a^m/+^* flies. (**E** + **G**) Shown are the 156 genes that were upregulated in bodies of *GBA1a^m/m^* and of *GBA1a^m/+^* flies (**E**; Enlargement of the upper cluster of panel **A**), and the 80 genes upregulated in heads (**G**; Enlargement of the upper cluster of panel **C**). (**F** + **H**) Gene ontology enrichment analysis of upregulated genes in bodies (**F**) and heads (**H**) of both homozygous (*GBA1a^m/m^*) and heterozygous (*GBA1a^m/+^*) flies in comparison to age matched controls. The hyper genomic test was used for the calculation of fold enrichment.

Transcriptomic analysis was performed on 12-day-old flies, to test whether inflammation and other related pathways are enriched in the *GBA1a^m/m^* flies. 449 genes were upregulated in bodies of *GBA1a^m/m^* flies, out of which 156 were upregulated in bodies of heterozygous flies as well (Figure 4A,B, Tables S1 and S2). 145 genes were upregulated in heads of the homozygous *GBA1a^m/m^* flies out of which 83 were also upregulated in heterozygous heads (Figure 4C,D). As predicted by the GO enrichment analysis tool (http://geneontology.org/) (The Gene Ontology Consortium, CC-BY 4.0) genes that were upregulated in *GBA1a^m/m^* and in *GBA1a^m/+^* flies presented enrichment of inflammation related pathways (Figure 4E,F). A slight elevation in inflammation related pathways was also noted in *GBA1a^m/m^* and *GBA1a^m/+^* heads (Figure 4G,H).

**Figure 5:**
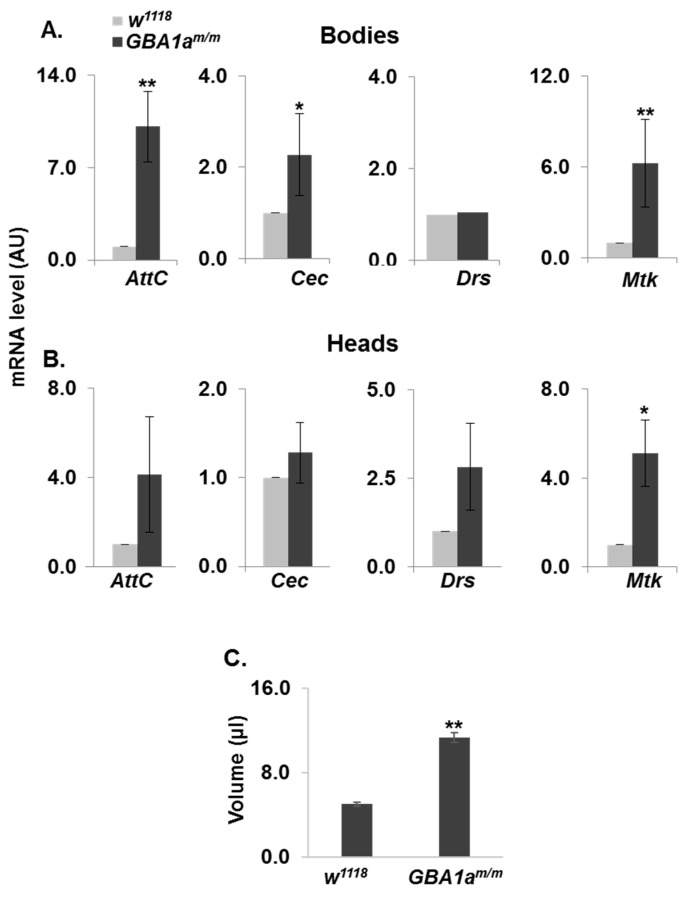
Activation of inflammation in *GBA1a^m/m^* flies. (**A** + **B**) mRNA levels of Attacin C (AttC), Cecropin (Cec), Drosomycin (Drs) and Metchnikowin (Mtk) in bodies (**A**) and heads (**B**) of *GBA1*^am/m^ and of age-matched controls (w^1118^) at 18 days post-eclosion, as analyzed by qRT-PCR. (**C**) A comparison between the volume of *GBA1a^m/m^* and age-matched controls body fluid, as measured by Hamilton syringe. Body fluid was extracted from 100 *GBA1*^am/m^ or age-matched control (w^1118^), 18-day-old females flies. The results in the different panels present the mean ± SEM of three-five independent experiments. * *p* < 0.05; ** *p* < 0.05.

To validate upregulation of inflammation in the *GBA1a^m/m^* mutant flies, we measured the levels of antimicrobial peptides (AMPs), which are known markers for innate immune response in *Drosophila* [35]. For that, we measured the mRNA levels of four AMPs by qRT-PCR analysis (Figure 5A,B). The results showed a significant elevation in three out of the four tested markers, in bodies of 18-day-old *GBA1a^m/m^* flies. Only Mtk level was significantly elevated in heads, which reflects a much lower degree of immune response in heads of *GBA1a^m/m^* flies, due to low expression level of *GBA1a* in heads. The volume of body fluids in 12-day-old mutant females and in age-matched controls was measured, since inflammation in flies is displayed by water retention [36], a phenotype that is more prominent in adult females than in adult males. The volume of *GBA1a^m/m^* body fluids was about twice that of age-matched normal females (Figure 5C), pointing to the presence of inflammation.

### 3.4. Enriched Pathways in GBA1a^m/m^ Flies

**Figure 6:**
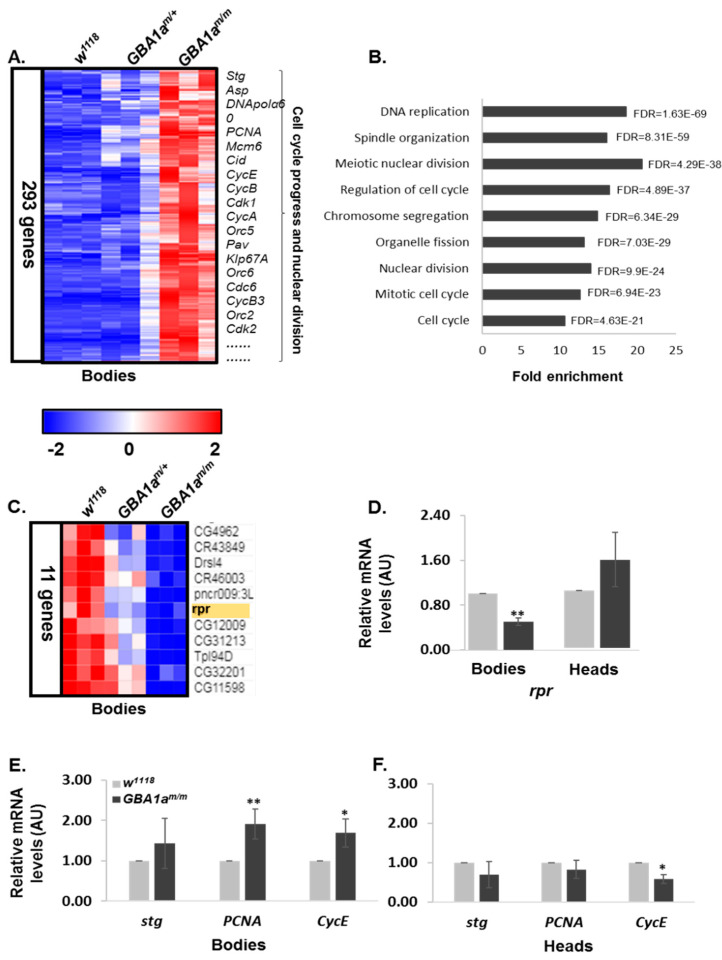
Cell cycle-related genes are upregulated in *GBA1a^m/m^* flies. (**A**) 293 genes were upregulated in bodies of *GBA1a^m/m^* flies in comparison to age-matched controls (*w^1118^*). (**B**) Gene ontology enrichment analysis of upregulated genes in bodies of *GBA1a^m/m^* flies in comparison to age-matched controls. The hyper genomic test was used for the calculation of fold enrichment. (**C**) Eleven genes were downregulated in bodies of 12-day-old *GBA1a^m/m^* flies with a slight reduction in *GBA1a^m/+^* flies, as predicted from transcriptomic analysis. *Reaper* (*rpr*) is highlighted in yellow. (**D**) mRNA level of the *Drosophila* apoptotic gene *reaper* (*rpr*) in bodies and in heads of *GBA1a^m/m^* and of control (*w^1118^*) flies, at 18 days post-eclosion, as analyzed by qRT-PCR. AU—Arbitrary units. (**E** + **F**) mRNA levels of the *Drosophila* cell cycle related genes markers: *string* (*stg*), proliferating cell nuclear antigen (*PCNA*) and *cyclin e* (*CycE*), in bodies (**E**) and in heads (**H**) of *GBA1a^m/m^* and control (*w^1118^*), flies at 18 days post-eclosion, as analyzed by qRT-PCR. The results in the different panels present the mean ± SEM of three-five independent experiments. * *p* < 0.05; ** *p* < 0.005.

As mentioned, transcriptomic analysis, performed on homozygous, heterozygous, and control flies revealed 449 genes that were upregulated in bodies of *GBA1a^m/m^* flies, out of which 156 genes were upregulated in heterozygous and 293 genes were exclusive to homozygous flies (Figure 4A,B, Figure 6A and Tables S3 and S4). High enrichment in pathways related to cell cycle progress and nuclear division was observed (Figure 6B). Eleven genes were downregulated in bodies, with a slight reduction in heterozygous flies as well (Figure 6C). Among these genes was reaper (rpr), a known PCD gene (Figure 6C) [27,37]. According to the gene ontology tool, and as expected, no significant pathway enrichment was noted in heads of the flies.

In order to validate the data, qRT-PCR analyses were performed with primers specific to reaper and to three markers of mitosis progression, which were upregulated according to the transcriptomic analyses: string (stg), proliferating cell nuclear antigen (PCNA), and cyclin e (CycE) [38,39]. The results revealed a significant reduction in the level of reaper and a significant elevation in levels of PCNA and CycE in bodies of *GBA1a^m/m^* flies (Figure 6D,E).

To summarize, our data strongly indicated enrichment in cell-cycle related genes in *GBA1a^m/m^* flies.

### 3.5. Delayed Midgut Morphogenesis in GBA1^m/m^ Flies 

**Figure 7:**
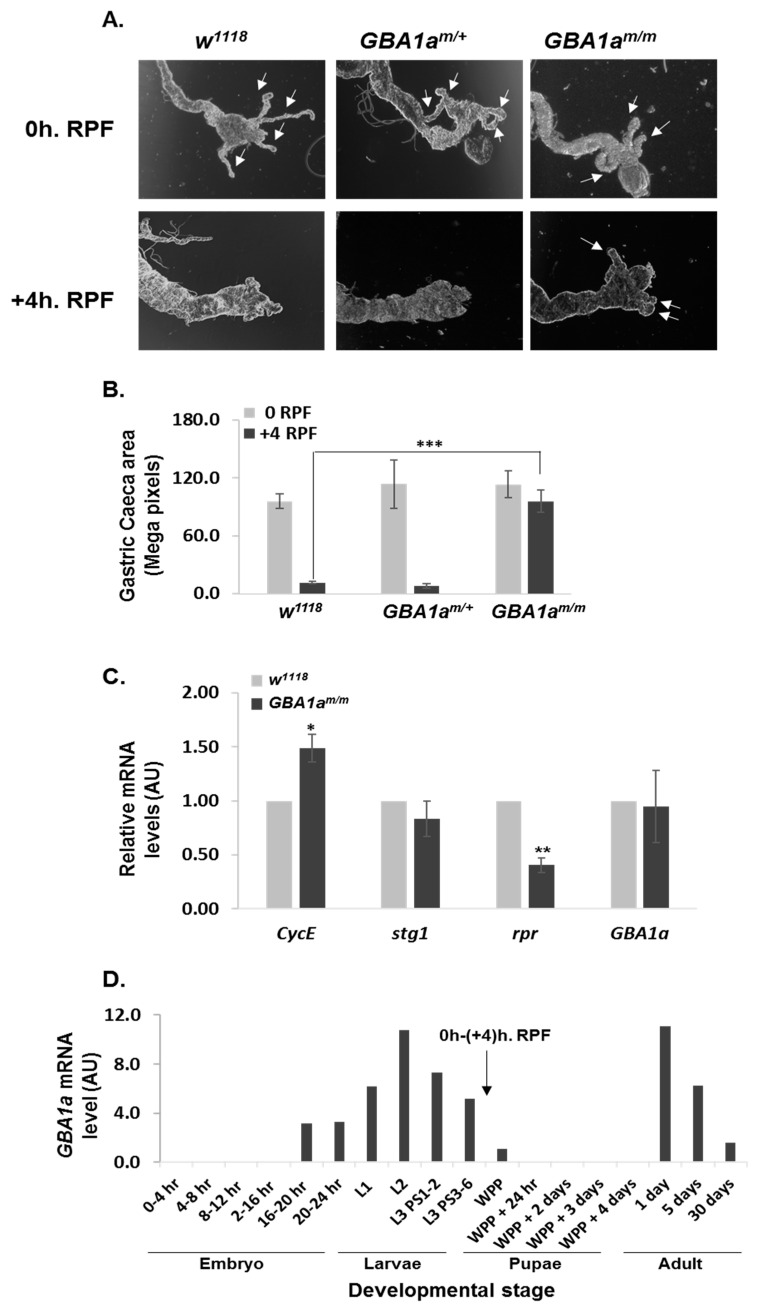
Midgut regression is delayed in *GBA1a^m/m^* flies. (**A**) Representing light microscopy images of midguts dissected from *GBA1a^m/m^*, *GBA1a^m/+^* and control (*w^1118^*) larvae at 0 h RPF (beginning of white pre pupa) and +4 h RPF. Arrows indicate gastric caeca. (**B**) Quantification of gastric caeca size at 0 and at +4 h RPF. Data represents mean ± SEM of 10–12 midguts per genotype. (**C**) mRNA levels of the *Drosophila* cell cycle related genes markers cyclin E (*CycE*), string (*stg*), the apoptotic gene *reaper* (*rpr*), and *GBA1a* in guts of *GBA1a^m/m^* and control (*w^1118^*) white pre pupae, as analyzed by qRT-PCR. Presented is the mean ± SEM of three independent experiments. (**D**) *GBA1a* mRNA expression according to FlyBase data (exonic expression by developmental stage). Relative puparium formation zero (0 h. RPF) and +four hours (+4 h. RPF) are marked with an arrow. WPP—white prepupa. * *p* < 0.05; ** *p* < 0.005; *** *p* < 0.001.

Since upregulation of cell cycle-related genes was observed in mutant *GBA1a^m/m^* flies, and based on the documented function of *GBA1a* in regulating autophagy-associated cell death during larval midgut development, resulting in midgut regression [8], we tested whether our mutant *GBA1a^m/m^* exhibits retarded death during larvae midgut development. To this end, we recorded the size of elongated gastric caeca, present at the junction of the foregut and midgut of mutant *GBA1a^m/m^* flies, of *GBA1a^m/+^* heterozygotes, as well as of age-matched controls (w^1118^), at the beginning of puparium (0 h. RPF) and four hours later (+4 h. RPF). While no significant change was obvious between the different lines at 0h. RPF, at +4 h. RPF, the area of gastric caeca of *GBA1a^m/m^* pupae was larger than that of control and of *GBA1a^m/+^* larvae (Figure 7A,B), pointing to a delay in midgut PCD in larvae of *GBA1a^m/m^* flies. Measurement of cell cycle-related genes in this developmental stage, employing qRT-PCR, revealed an increase in CycE mRNA level (Figure 7C), but no change in the level of stg was found for adults (Figure 6E,F) and not in that of PCNA (Figure S1), differently from what was shown in bodies of adult flies (Figure 6E,F). This variation in PCNA mRNA levels may reflect a different expression pattern of the tested genes between midgut and adult bodies. There was also a decrease in rpr mRNA level (Figure 7C), as seen in bodies of 18-day-old *GBA1a^m/m^* flies (see Figure 6D).

Retarded midgut development was in agreement with the mRNA expression of *GBA1a* during *Drosophila* development, which is absent in the embryo and pupae and is the highest during larvae stages and early adulthood (FlyBase.org) (Figure 7D).

### 3.6. Longevity and Locomotion of GBA1a^m/m^ Flies

Since there was delayed midgut development in *GBA1a^m/m^* larvae, it is conceivable that other apoptotic related processes are defective, which may delay normal development, locomotion, and longevity of the *GBA1a^m/m^* flies. To this end, we followed different developmental stages in mutant and age-matched control flies. No significant change was recorded in egg laying or in larvae hatching between *GBA1a^m/m^* flies and their age-matched controls (Table 3).

**Figure 8:**
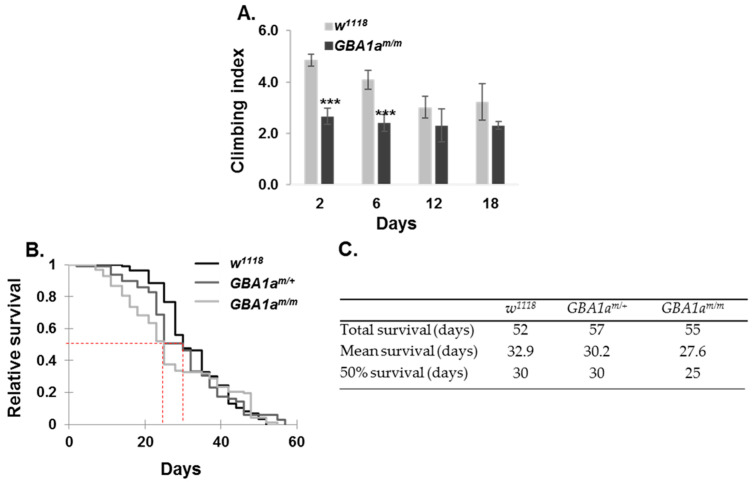
Phenotype of *GBA1a^m/m^* flies. (**A**) Climbing assay comparing the locomotion of thirty *GBA1a^m/m^* and of thirty control (*w^1118^*) flies at different time points. Presented is the average of four different experiments ± SEM. *** *p* < 0.005. (**B**) Kaplan Meier survival curve showing longevity of *GBA1a^m/m^*, of *GBA1a^m/+^*, and of control (*w^1118^*) flies at 25 °C. (**C**) A table showing the total, mean, and 50% survival of *GBA1a^m/m^*, *GBA1a^m/+^*, and of control (*w^1118^*) flies, as obtained from Kaplan Meier analysis.

Next, percent of larvae surviving to pupae and percent of pupae surviving to adult was tested in *GBA1a^m/m^* in comparison to that in heterozygous and in age-matched control lines, at 25 °C and 29 °C. A significant reduction in survival was observed for the *GBA1a^m/m^* larvae surviving to pupae at 25 °C and 29 °C (Table 4). No significant difference was recorded for percent homozygous pupae surviving to adult. A reduction in the negative geotaxis of *GBA1a^m/m^* flies was noted at day 2 and 6 post-eclosion, with no further changes, in comparison to that of age-matched controls (Figure 8A). This decreased locomotion is in agreement with the peak of expression of *GBA1a* in the first days after eclosion (Figure 7C). Survival of *GBA1a^m/m^* was also decreased during early adulthood, with 50% survival of the *GBA1a^m/m^* flies reached five days earlier than that of the control flies (w^1118^), (Figure 8B,C). However, the overall survival of the *GBA1a^m/m^* flies did not significantly differ from that of the age-matched controls (Figure 8B,C). These results also reflect the highest expression of *GBA1a* in the first days post-eclosion (Figure 7C).

Taken together, our results point to developmental defects at pre-pupation and in young adult *GBA1a^m/m^* flies, which affect their normal maturation, as well as their locomotion and is reflected in an abnormal survival, compared to that of age-matched controls.

## 4. Discussion

In the present study, we searched for a possible function of the *GBA1a*-encoded protein. Though 47% identical and 80% similar to the *GBA1b*-encoded GCase, the *GBA1a*-encoded protein has no lysosomal GCase activity. It does not have activity of other known lysosomal glucosidases. We were able to show that the mutant protein underwent ERAD and activated UPR. Interestingly, we also recorded inflammation in the *GBA1a^m/m^* flies. Since we did not find any substrate accumulation [5], we assumed that this is a UPR induced inflammation, well documented in the literature [36,37,38]. Thus, it was demonstrated that *ATF4* and *Xbp1*, two branches of the UPR, are essential transcription factors of the inflammatory cytokines IL8, IL6, and MCP1 in human aortic endothelial cells [33]. Likewise, hyper-activated IRE1α, another UPR-related gene, was found as an activator of the NLRP3 inflammasome [32] and as an activator of JNK and NF-κB, which induce the production of inflammatory cytokines in INS-1 cells [34]. UPR-induced inflammation was documented in different diseases, including Alzheimer disease [40], diabetes [41], and atherosclerosis [42].

In addition to the upregulation in inflammatory related genes in the *GBA1a^m/m^* flies, transcriptomic analysis data revealed a high enrichment in cell cycle and nuclear division pathways. Validation with qRT-PCR analysis showed elevation in *PCNA* and *cyclin E* mRNAs, known markers for mitosis in *Drosophila*, as well as downregulation in *reaper*, a known apoptosis-related gene [27,37].

In the last decade, autophagic-induced cell death, autosis, was documented [43]. In an effort to isolate autosis-related genes, resveratrol-treated A549 human lung carcinoma cells were transfected with a RNAi library, and colonies with significantly longer survival were tested for the nature of the transfected RNAis. *GBA1* was identified as an autosis regulator [44]. In order to establish the function of *GBA1* in autosis, and knowing that there is autosis during fly larvae midgut maturation, where there is high expression level of *GBA1a*, the authors analyzed the effect of downregulation in *Drosophila* using *GBA1a* RNAi. The authors documented a regression of larval midgut caeca in *GBA1a* KD [8]. We could recapitulate these results using the *GBA1a^m/m^* flies. We also tested the effect of the mutant protein on the ability of the larvae and pupae to mature. Our results showed that the mutant *GBA1a*-encoded protein affected larvae survival. Interestingly the effect of the mutation corresponded well to the time of high expression of the *GBA1a* gene during development. *GBA1a* expression has two peaks (FlyBase.org, see Figure 7C): during larvae stages 2–3 and during the first days post-eclosion. These are the two time points in which defects were observed in the *GBA1a^m/m^* flies: midgut regression in the larvae, and locomotion deficit and decreased survival in the first days post-eclosion.

From previous reports [3,4,6,7] and from our results [5], it seems that *GBA1a*-encoded protein functions differently from its closely related *GBA1b*-encoded protein. While the *GBA1b* encodes a *bona fide* GCase, *GBA1a* encodes a protein with no GCase activity, but with a function during terminal apoptotic stages of development. Interestingly, the one human active *GBA1* gene possess these two activities [44]. Likewise, in mice, there is one *GBA1* gene, which encodes a *bona fide* GCase and seems to be involved in cell death, cell differentiation, cell proliferation, signaling, and system development (http://www.informatics.jax.org/marker/MGI:95665, Gene Ontology classification).

The phenomenon of proteins with more than one function, designated protein moonlighting, is well-recognized in the literature [45]. Cathepsin L, a lysosomal protein, is involved in the initiation of protein degradation and turnover of plasma membrane proteins for maintenance of intestinal homeostasis. In addition, it enters the nucleus and accelerates cell cycle progression [46]. Cytochrome C functions in the electron transport chain in mitochondria. However, it also functions as a proapoptotic mediator [47]. Another moonlighting protein is the endocytic protein EHD2. EHD2 is a plasma membrane-associated protein that regulates internalization. It contains a nuclear localization sequence, which enables its shuttling to the nucleus, where it functions as a transcription repressor [10].

To summarize, our results confirm that the *GBA1a*-encoded protein mediates larvae mid-gut regression. Its mutant variant (*GBA1a^m^*) activates UPR, which leads to its ERAD, evokes the inflammatory response, and results in deregulated development, attenuated locomotion performance, and a change in the survival of the flies.

## Figures and Tables

**Table 1:** Active site associated amino acids in human and fly *GBA1*-encoded proteins.

Role in Active Site	Human GCase	*GBA1a* Encoded	*GBA1b* Encoded
Catalytic amino acid	E235	E298	E298
Catalytic amino acid	E340	E405	E405
Stabilizing the substrate	F128	F194	F194
Stabilizing the substrate	W179	W246	W246
Stabilizing the substrate	F246	F308	F308
Stabilizing the substrate	Y313	Y389	Y389
Stabilizing the substrate	W381	W408	W408
Stabilizing the substrate	F397	---	F463

**Table 2:** Primers used in the present study. The table depicts all primers used in the present study for qRT-PCR analyses.

Name	Primer Sequence
*ATF4*	F: 5′-AGACGCTGCTTCGCTTCCTTC-3′
	R: 5′-GCCCGTAAGTGCGAGTACGCT-3′
*Hsc70* *-3*	F: 5′-GCTGGTGTTATTGCCGGTCTGC-3′
	R: 5′-GATGCCTCGGGATGGTTCCTTGC-3′
s*XBP1*	F: 5′-CCGAACTGAAGCAGCAACAGC-3′
	R: 5′-GTATACCCTGCGGCAGATCC-3′
*AttC*	F: 5′-CTGCACTGGACTACTCCCACATCA-3′
	R: 5′-CGATCCTGCGACTGCCAAAGATTG-3′
*CEC*	F: 5′-CATTGGACAATCGGAAGCTGGGTG-3′
	R: 5′-TAATCATCGTGGTCAACCTCGGGC-3′
*DRS*	F: 5′-AGTACTTGTTCGCCCTCTTCGCTG-3′
	R: 5′-CCTTGTATCTTCCGGACAGGCAGT-3′
*MTK*	F: 5′-CATCAATCAATTCCCGCCACCGAG-3′
	R: 5′-AAATGGGTCCCTGGTGACGATGAG-3′
*stg*	F: 5’-CGTCGTCGAGTCAACAGCTCTTC-3′
	R: 5′-GTATTTCGGAGTGTGGTTGTGCG-3′
*PCNA*	F: 5’-GCAGCGACTCCGGCATTCAG-3′
	R: 5′-CGCAGGGTCAGCGAGACAAG-3′
*R*	F: 5’-GCTGGATGGAGCCATTCTTCCG-3′
	R: 5′-CCTGGGCCATAAGCACTTCGTC-3′
*RPR*	F: 5’CATACCCGATCAGGCGACTC-3′
	R: 5’-GCTTGCGATATTTGCCGGAC-3′
*rp49*	F: 5′-TAAGAAGCGCACAAAGCACT-3′
	R: 5′-GGGCATCAGATATTGTCCCT-3′

**Table 3:** Egg laying and larval hatching.

Temperature	Genotype	Number of Laid Eggs	Number of Hatching
25 °C	*w^1118^*	105	72
	*GBA1a^m/m^*	103	64
		*p* = 0.61	*p* = 0.35

Number of egg laying and larval hatching of w^1118^ and *GBA1a^m/m^* lines at 25 °C. *p* values are shown below the relevant numbers.

**Table 4:** Survival at different developmental stages.

Temperature	Genotype	% Larvae Surviving to Pupae	% Pupae Surviving to Adult
25 °C	*w^1118^*	100	94
	*GBA1a^m/+^*	98.1	92
	*GBA1a^m/m^*	90 *	87
29 °C	*w^1118^*	96.9	85.6
	*GBA1a^m/+^*	95.2	90.3
	*GBA1a^m/m^*	86.7 *	80.6

Survival of *GBA1a^m/m^*, *GBA1a^m/+^*, and w^1118^ larvae to pupae and of pupae to adult flies at 25 and 29 °C. * *p* < 0.05.

## Data Availability

The data will be available on December 2022 at https://www.ncbi.nlm.nih.gov/geo/query/acc.cgi?acc=GSE142144 (ID GSE142144).

## Supplementary Materials

The following are available online at https://www.mdpi.com/2073-4409/10/3/630/s1. Figure S1. mRNA level of *Drosophila PCNA*; Table S1. Upregulated genes in bodies of *GBA1a^m/m^* and in *GBA1a^m/+^*. Table S2. GO enrichment analysis of upregulated genes in bodies of *GBA1a^m/m^* and in *GBA1a^m/+^;* Table S3. Upregulated genes in bodies of *GBA1a^m/m^*; Table S4. GO enrichment analysis of upregulated genes in bodies of *GBA1a^m/m^*.
