# Supplementary material for: The Uncovered Function of the Drosophila GBA1a-Encoded Protein
Source: Cells. 2021 Mar 12;10(3):630. doi: 10.3390/cells10030630 (PMC8000066; doi:10.3390/cells10030630)
Supplement: Supplementary file 1 [file cells-10-00630-s001.pdf]

Supplementary Materials

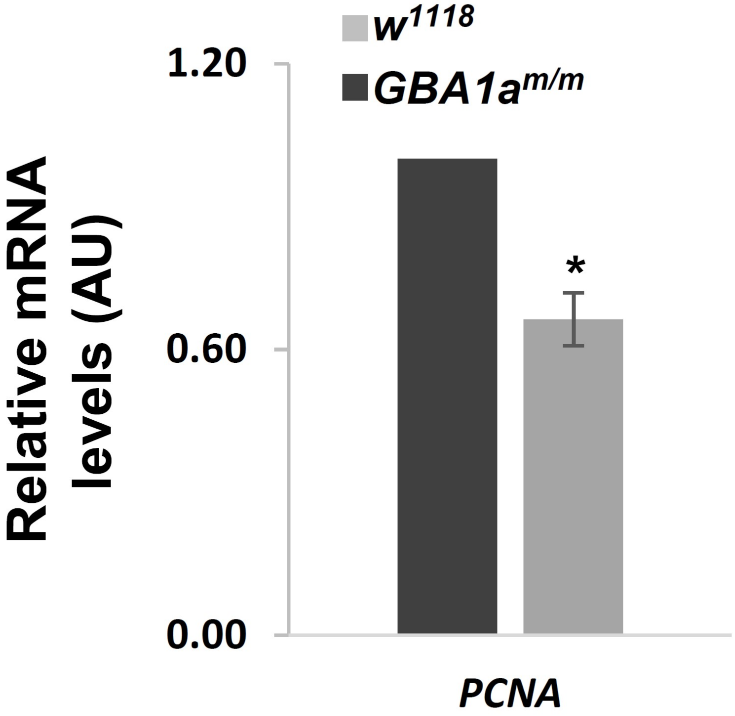

Figure S1. mRNA level of *Drosophila PCNA*. mRNA levels of the *Drosophila* cell cycle gene Proliferating cell nuclear antigen (*PCNA*) in guts of *GBA1a*$^{m/m}$ and control (*w*^{1118}) white pre pupae, as analyzed by qRT-PCR. Presented is the mean ± SEM of three independent experiments.

Table S1. Upregulated genes in bodies of $GBA1a^{m/m}$ and in $GBA1a^{m/+}$.

A.

1	IM14	54	CG5867	107	NimB4
2	PGRP-SC2	55	Jhl-21	108	CG16965
3	CG17047	56	CDase	109	CG33926
4	CG4734	57	CG33296	110	CG42807
5	CG7896	58	CG31706	111	PGRP-SD
6	CG10062	59	Hexo2	112	CG34291
7	CG11550	60	CG17374	113	P5CDh2
8	CG10232	61	Cyp6a23	114	SPH93
9	CG31809	62	sl	115	CG9747
10	CG40198	63	wtrw	116	CG16904
11	Cyp6a8	64	wat	117	CG6675
12	CG15695	65	CG11841	118	CG5757
13	htl	66	CG3097	119	CG9312
14	CG32260	67	CG1732	120	CG32364
15	CG14946	68	Drs	121	CG43291
16	Cpr11B	69	Dro	122	Spn42De
17	CG4908	70	CG9416	123	CG17107
18	CG14034	71	CG6225	124	CG30384
19	CecA1	72	CG40486	125	Cyp313a4
20	Mtk	73	CG1236	126	AstC-R1
21	CecB	74	vis	127	mthl8
22	AdoR	75	CG18136	128	CG6654
23	CG11951	76	Tsp5D	129	CR45973
24	CR45822	77	CG4115	130	mth
25	CecC	78	CG9394	131	CR45835
26	Cyp305a1	79	CG8745	132	Zip71B
27	CG8925	80	CG9498	133	CG10514
28	CG30339	81	GstD3	134	CG13931
29	CG6362	82	lectin-28C	135	CG2064
30	Aph-4	83	CG4098	136	Pvf2
31	wrapper	84	Cyp4g15	137	CG14642
32	CG14798	85	mst	138	SPH93
33	CG4409	86	AttC	139	CG33177
34	Dhc98D	87	eco	140	CG18563
35	CG40470	88	GstD2	141	Obp99d
36	Top3alpha	89	CR46035	142	Obp69a
37	CG1537	90	CG13978	143	Ugt35b
38	alrm	91	CG1924	144	CS-2
39	gb	92	CG6688	145	CG18067
40	CG14855	93	He	146	CG10513
41	CG13742	94	CG17167	147	Cyp4e1
42	Cyp4e3	95	Cyp6d2	148	CG31817
43	CG11686	96	CG30154	149	Cyp4g1
44	CG15209	97	CG15347	150	CG44072
45	CG30285	98	CG17855	151	Elo68alpha
46	Trf	99	CG15536	152	scpr-C
47	CG31028	100	CG1941	153	upd2
48	Coprox	101	CR13130	154	CG3919
49	CG15546	102	CR43855	155	Or67d
50	Sod3	103	CR44855	156	Or45a
51	Cnx14D	104	Idgf3		
52	fng	105	mre11		
53	CG6951	106	CG6753		

B.

Biological Process	Known Genes (D. Mel.)	Expected	Observed	Fold Enrichment	FDR
Response to stimulus	2503	11.62	30	2.58	1.19E-04
Defense response to other organism	327	1.52	10	6.58	7.89E-03
Oxidation-reduction process	573	2.66	13	4.89	8.66E-03
Defense response	345	1.6	10	6.24	9.4E-03
Response to biotic stimulus	381	1.77	10	5.65	1.10E-02
Interspecies interaction between organisms	387	1.8	10	5.65	1.10E-02
Response to other organism	381	1.77	10	5.65	1.46E-02
Immune system process	290	1.35	8	5.94	4.27E-02

A. List of upregulated genes in bodies of 12 day sold $GBA1a^{m/m}$ and $GBA1a^{m/+}$ flies compared to age matched controls, as analyzed by RNA-seq by cutoffs pFDR < 0.05 and fold-change difference. 2.The genes appear at the same order as they appear in the hierarchical clustering, structured by the Morpheus tool (https://software.broadinstitute.org/morpheus/). Genes that are related to immune response in the fly are highlighted in blue. **B.** Gene ontology enrichment analysis of upregulated genes in bodies of homozygous ($GBA1a^{m/m}$) and heterozygous ($GBA1a^{m/+}$) flies in comparison to age matched controls. Hypergenomic test was used for the calculation off old enrichment.

Table S2. GO enrichment analysis of upregulated genes in bodies of *GBA1a*$^{m/m}$ and in *GBA1a*$^{m/+}$

Biological Process	Known Genes (D. Mel.)	Expected	Observed	Fold Enrichment	FDR
Response to stimulus	2503	11.62	30	2.58	1.19E-04
Defense response to other organism	327	1.52	10	6.58	7.89E-03
Oxidation-reduction process	573	2.66	13	4.89	8.66E-03
Defense response	345	1.6	10	6.24	9.4E-03
Response to biotic stimulus	381	1.77	10	5.65	1.10E-02
Interspecies interaction between organisms	387	1.8	10	5.65	1.10E-02
Response to other organism	381	1.77	10	5.65	1.46E-02
Immune system process	290	1.35	8	5.94	4.27E-02

Gene ontology enrichment analysis of upregulated genes in bodies of homozygous (*GBA1am/m*) and heterozygous (*GBA1a*$^{m/+}$*)* flies in comparison to age matched controls. Hyper-genomic test was used for the calculation of fold enrichment.

Table S3. Upregulated genes in bodies of *GBA1a* $^{m/m}$.

A.

**1**	CG12708	**54**	CG11263	**107**	CG7730	**160**	CG4991	**213**	Cyp6a19	**266**	Incenp
**2**	CG15506	**55**	CG6967	**108**	CG12288	**161**	CG13741	**214**	Chd3	**267**	Klp61F
**3**	CG9192	**56**	Nse1	**109**	CG15820	**162**	Msh6	**215**	CG14814	**268**	mad2
**4**	CG31272	**57**	HP1D3csd	**110**	Fancl	**163**	bond	**216**	Orc2	**269**	cad
**5**	CR32773	**58**	CG17129	**111**	BoYb	**164**	yin	**217**	asp	**270**	CG9925
**6**	CG15784	**59**	CG5780	**112**	squ	**165**	CG14218	**218**	Mcm5	**271**	Klp67A
**7**	Bin1	**60**	CG15629	**113**	pim	**166**	DNApol-alpha73	**219**	fs(1)Ya	**272**	dup
**8**	pst	**61**	CG7352	**114**	thr	**167**	Spc25	**220**	Klp3A	**273**	tum
**9**	CG34227	**62**	CG2930	**115**	Trf4-2	**168**	CG4476	**221**	RnrS	**274**	wisp
**10**	Nxf3	**63**	fal	**116**	CG13599	**169**	Ctf4	**222**	spn-E	**275**	Caf1-105
**11**	Arc2	**64**	CG17574	**117**	CG15047	**170**	Nup43	**223**	CG2990	**276**	CG9926
**12**	Rel	**65**	CG7888	**118**	Cap-D2	**171**	gammaTub37C	**224**	Nek2	**277**	CG3457
**13**	CG1667	**66**	GILT3	**119**	msd1	**172**	Spc105R	**225**	feo	**278**	Rbf2
**14**	CG4269	**67**	CG4686	**120**	BthD	**173**	DNApol-epsilon58	**226**	CG11674	**279**	Ote
**15**	Zip99C	**68**	CR44291	**121**	Mcm7	**174**	dpa	**227**	zpg	**280**	polo
**16**	CG1942	**69**	CG8012	**122**	Cdc6	**175**	Ts	**228**	AGO3	**281**	spd-2
**17**	Tep1	**70**	CG9512	**123**	msb1l	**176**	DNApol-alpha180	**229**	aurA	**282**	CycE
**18**	CG18557	**71**	Hml	**124**	aurB	**177**	Poc1	**230**	piwi	**283**	exu
**19**	Tsp2A	**72**	Ance-3	**125**	Cdk1	**178**	CG1503	**231**	barr	**284**	nos
**20**	Ir76a	**73**	CG12112	**126**	bora	**179**	CG7497	**232**	vas	**285**	cmet
**21**	Tsp42Ed	**74**	fs(1)M3	**127**	sip2	**180**	Apc2	**233**	CG17018	**286**	rpk
**22**	yellow-g	**75**	GILT1	**128**	CG7372	**181**	ball	**234**	CycB	**287**	Mink
**23**	alphaTub85E	**76**	CG2982	**129**	CycB3	**182**	CG12702	**235**	CycA	**288**	qua
**24**	AOX4	**77**	CG14036	**130**	dah	**183**	Cks30A	**236**	BigH1	**289**	sub
**25**	vret	**78**	rhi	**131**	borr	**184**	CG9752	**237**	Hsp27	**290**	twe
**26**	CG31875	**79**	CG8223	**132**	fzy	**185**	CG5235	**238**	gwl	**291**	Orc6
**27**	GstE7	**80**	CG9519	**133**	RpS5b	**186**	Orc5	**239**	gnu	**292**	tor
**28**	CG6733	**81**	CG7777	**134**	Mcm3	**187**	CG31898	**240**	swa	**293**	CG32625
**29**	obst-B	**82**	cid	**135**	ncd	**188**	CG16978	**241**	aub		
**30**	CG15169	**83**	Ugt86Da	**136**	PCNA	**189**	CG11164	**242**	dnk		
**31**	CR43144	**84**	dgt6	**137**	CG13690	**190**	CG17027	**243**	Orc1		
**32**	Strica	**85**	Corp	**138**	CG6425	**191**	spirit	**244**	Zwilch		
**33**	CG17324	**86**	Fen1	**139**	CG4586	**192**	nopo	**245**	CG14931		
**34**	CR45435	**87**	phr6-4	**140**	mud	**193**	Cdk2	**246**	Mcm2		
**35**	CG14339	**88**	CG4950	**141**	mei-38	**194**	Grip71	**247**	otu		
**36**	CG17658	**89**	CG6074	**142**	Claspin	**195**	Rab23	**248**	APC7		
**37**	CG30401	**90**	CG9759	**143**	CG8478	**196**	CG43093	**249**	Hsp26		
**38**	CG13046	**91**	Act42A	**144**	scra	**197**	CG6409	**250**	mtrm		
**39**	nmdyn-D7	**92**	mus301	**145**	Mcm10	**198**	CG7695	**251**	lok		
**40**	Cyp312a1	**93**	Sdr	**146**	Rif1	**199**	Cas	**252**	osk		
**41**	dmGlut	**94**	Cht2	**147**	sti	**200**	png	**253**	stg		
**42**	btl	**95**	asl	**148**	zuc	**201**	CR43836	**254**	elg1		
**43**	CG8303	**96**	yellow-c	**149**	cav	**202**	mos	**255**	pav		
**44**	CG9377	**97**	CG13950	**150**	CBP	**203**	krimp	**256**	CG5194		
**45**	CG5111	**98**	Sas-4	**151**	jumu	**204**	pgc	**257**	DNApol-alpha60		
**46**	CR43087	**99**	ImpL1	**152**	CG10336	**205**	CG14516	**258**	Ndc80		
**47**	fs(1)N	**100**	ana2	**153**	cort	**206**	CG18190	**259**	Mcm6		
**48**	mms4	**101**	bcd	**154**	Nuf2	**207**	CG2187	**260**	me31B		
**49**	CG34265	**102**	CG18586	**155**	pbl	**208**	Duox	**261**	WRNexo		
**50**	CG34456	**103**	CG8420	**156**	gd	**209**	CG6927	**262**	Ggt-1		
**51**	ana3	**104**	CG7208	**157**	stil	**210**	dhd	**263**	tos		
**52**	CG10357	**105**	CG3679	**158**	sofe	**211**	Lds	**264**	RnrL		
**53**	CG14545	**106**	CG44005	**159**	CG13841	**212**	alphaTub67C	**265**	Caf1-180		

B.

Biological Process	Known Genes (D. Mel.)	Expected	Observed	Fold Enrichment	FDR
Cell cycle	616	9.7	99	10.21	2.87E-66
Mitotic cell cycle	411	6.47	78	12.05	3.72E-55
Nuclear division	242	3.81	50	13.12	4.92E-35
Organelle fission	256	4.03	50	12.4	4.47E-34
Reproductive process	1174	18.49	86	4.65	8.65E-32
Cellular component organization	2608	41.07	121	2.95	6.27E-33
Sexual reproduction	1041	16.37	77	4.7	3.34E-28
DNA replication	91	1.43	28	19.54	4.23E-23
Spindle organization	119	1.87	29	15.48	1.42E-21
Meiotic nuclear division	162	2.55	29	11.37	1.86E-18

A. List of upregulated genes exclusive to bodies of 12 day sold *GBA1a* $^{m/m}$ compared to age matched controls, as analyzed by RNA-seq, by cutoffs pFDR < 0.05 and fold-change difference. 2.The genes appear at the same order as they appear in the hierarchical clustering, structured by the Morpheus tool (https://software.broadinstitute.org/morpheus/). Genes that are related to cell cycle progress and nuclear division in the fly are highlighted in green. **B.** Gene ontology enrichment analysis of upregulated genes in bodies of homozygous (*GBA1a* $^{m/m}$) flies in comparison to age matched controls. Hypergenomic test was used for the calculation off old enrichment.

Table S4. GO enrichment analysis of upregulated genes in bodies of *GBA1a$_{m/m}$*.

Biological Process	Known Genes (D. Mel.)	Expected	Observed	Fold Enrichment	FDR
Cell cycle	616	9.7	99	10.21	2.87E-66
Mitotic cell cycle	411	6.47	78	12.05	3.72E-55
Nuclear division	242	3.81	50	13.12	4.92E-35
Organelle fission	256	4.03	50	12.4	4.47E-34
Reproductive process	1174	18.49	86	4.65	8.65E-32
Cellular component organization	2608	41.07	121	2.95	6.27E-33
Sexual reproduction	1041	16.37	77	4.7	3.34E-28
DNA replication	91	1.43	28	19.54	4.23E-23
Spindle organization	119	1.87	29	15.48	1.42E-21
Meiotic nuclear division	162	2.55	29	11.37	1.86E-18

Gene ontology enrichment analysis of upregulated genes in bodies of homozygous (*GBA1a$^{m/m}$*) flies in comparison to age matched controls. Hyper-genomic test was used for the calculation of fold enrichment.
